# Supplementary material for: Association between oral microbiome and seven types of cancers in East Asian population: a two-sample Mendelian randomization analysis
Source: Front Mol Biosci. 2023 Nov 21;10:1327893. doi: 10.3389/fmolb.2023.1327893 (PMC10702768; doi:10.3389/fmolb.2023.1327893)
Supplement: Supplementary file 1 [file DataSheet1.ZIP › Supplementary Materials/MR plots for tongue/tongue═╝/Prostate cancer/pheno.2037_to_prostate cancer_leave_one_out.pdf]

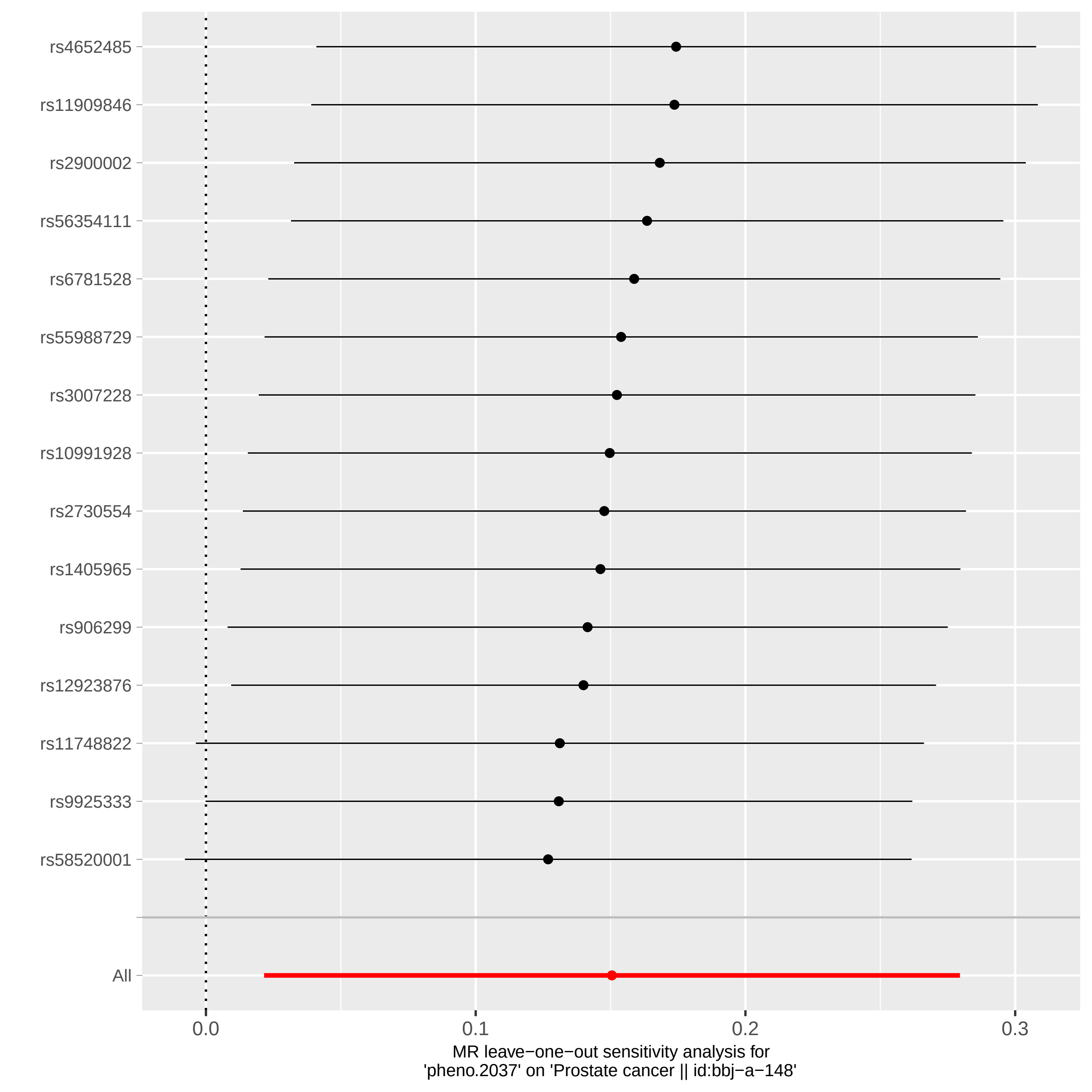
rs4652485
rs11909846
rs2900002
rs56354111
rs6781528
rs55988729
rs3007228
rs10991928
rs2730554
rs1405965
rs906299
rs12923876
rs11748822
rs9925333
rs58520001
All
0.0
0.1
0.2
0.3
MR leave−one−out sensitivity analysis for
'pheno.2037' on 'Prostate cancer || id:bbj−a−148'
